# District of Columbia and the Vice Problem

**Published:** 1914-06

**Authors:** 


### District of Columbia and the Vice Problem.

At this time when many cities are individually striving to solve the vice problem it is with interest that we watch the outcome of any new procedure. Of special moment is the operation of the Kenyon Injunction and Abatement Law, passed by the District of Columbia. With reference to this law the *Bulletin of the American Social Hygiene Association* has to say:

"The Kenyon Injunction and Abatement Law, based upon the Iowa law and in substantial conformity with it, and designed to furnish a ready means for the suppression of houses of prostitution in the District of Columbia, was signed by President Wilson February 7, 1914. The President retained the bill from its passage on January 26th until February 7th, in order to give opportunity to raise funds and provide means to care for the women that would be driven by it out of the segregated district known as "The Division." For this purpose a Citizens' Committee of fifty was appointed, and on March 26th it appears that their work was practically complete.

"From investigation by a representative of the American Social Hygiene Association, it appears that the houses closed their doors without court action, and as a result of the public sentiment brought to bear upon the public officials charged with the maintenance of decency and good order. The Citizens' Committee rendered assistance to forty-three of the fifty-three former inmates of the district who came under its supervision. Three of the women have been restored to the homes of relatives and friends, three have received medical care, one is under institutional care, employment has been obtained for nine, twenty-two are under the friendly supervision of others, but the whereabouts of the remaining eight are unknown. How many prostitutes may have scattered to other portions of the city and renewed their trade in tenement houses or apartments, or through other agencies, is not yet determined, but an effective effort to follow up the enforcement of this new law seems to be assured.

"It is of interest in considering the effects of this law that Alexandria, in Virginia, has found it necessary to appoint a committee to consider ways and means of combating the influx of prostitutes since the Kenyon law became effective."

